# A New Raman Metric for the Characterisation of Graphene oxide and its Derivatives

**DOI:** 10.1038/srep19491

**Published:** 2016-01-18

**Authors:** Alice A. K. King, Benjamin R. Davies, Nikan Noorbehesht, Peter Newman, Tamara L. Church, Andrew T. Harris, Joselito M. Razal, Andrew I. Minett

**Affiliations:** 1 Laboratory for Sustainable Technology, Department of Chemical and Biomolecular Engineering, University of Sydney, NSW, 2006, Australia; 2 Department of Physics, University of Surrey, Guildford, GU2 7XH, UK; 3 Biomaterials and Tissue Engineering Research Unit, University of Sydney, NSW, 2006, Australia; 4 ARC Future Fellow, Institute for Frontier Materials, Deakin University, Geelong, Australia; 5 Australian Institute for NanoScience and Technology (AINST), University of Sydney, NSW, 2006

## Abstract

Raman spectroscopy is among the primary techniques for the characterisation of graphene materials, as it provides insights into the quality of measured graphenes including their structure and conductivity as well as the presence of dopants. However, our ability to draw conclusions based on such spectra is limited by a lack of understanding regarding the origins of the peaks. Consequently, traditional characterisation techniques, which estimate the quality of the graphene material using the intensity ratio between the D and the G peaks, are unreliable for both GO and rGO. Herein we reanalyse the Raman spectra of graphenes and show that traditional methods rely upon an apparent G peak which is in fact a superposition of the G and D’ peaks. We use this understanding to develop a new Raman characterisation method for graphenes that considers the D’ peak by using its overtone the 2D’. We demonstrate the superiority and consistency of this method for calculating the oxygen content of graphenes, and use the relationship between the D’ peak and graphene quality to define three regimes. This has important implications for purification techniques because, once GO is reduced beyond a critical threshold, further reduction offers limited gain in conductivity.

Graphene has become a primary research focus in many current fields of science as evidenced by the recent announcement from the European Commission of €1 billion funding for the commercialisation of graphene research over the next ten years^1^. This, coupled with the recent proliferation of publications on graphene and graphene oxide (GO)^2^,^3^,^4^, suggests that research on 2-D graphitic materials will increase. Raman spectroscopy has proven to be one of the most powerful techniques for the characterisation of graphene, as its phonon modes provide explicit insights into changes in layer structure, dopants, conductivity etc^5^,^6^. For practical reasons, materials scientists and engineers developing devices and larger-scale materials often work with GO and reduced graphene oxide (rGO), which are easier to process due to improved solubility, better control over size and more scalable processing methods^7^,^8^,^9^,^10^,^11^,^12^. Unfortunately the tools that are used to extract physicochemical information from the Raman spectra of graphenes have not proven directly applicable to these materials. In particular, the I_D_/I_G_ ratio, which has been validated as a measure of inter-defect distance in graphene, is unreliable when applied to GO and rGO^13^,^14^,^15^,^16^. Ferrari and Robertson defined an amorphisation trajectory, in which the I_D_/I_G_ ratio of amorphous carbon (that was still sp^2^ bonded) would increase with the removal of defects, proportional to the square of the crystallite size^17^. In this way they define a transition between carbons that have a crystallite size smaller than ~2 nm, which should obey the Ferrari–Robertson relation, and those with larger crystallites, which obey the Tuinstra–Koenig relation. The discontinuity in the ratio-to-structure relationship is attributed to the complete distortion of the aromaticity at very high defect densities; however, this proposal has been vitiated for GO and rGO as high-resolution STM imaging has shown areas of aromaticity up to 8 nm^2^ even in raw GO^18^.

## Results and Discussion

**Figure 1:**
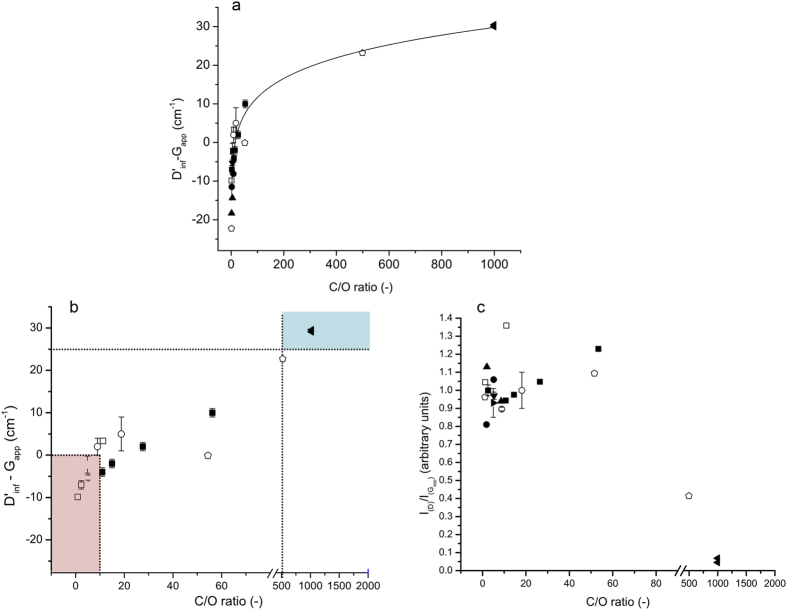
Changes in Raman features as a function of C/O atom ratio. (**a**) Difference in the D’_inf_ position and G_app_ as a function of the C/O ratio for multiple samples from various sources; with line of best fit. (**b**) Change in D’_inf_ − G_app_ as a function of C/O ratio on a broken scale for clarity, coloured boxes show the regions of GO (red), and graphene (blue), with rGO occupying the region in between, standard error in the mean used for samples measured in our laboratory, thermally reduced between 0 and 1,000 °C (∎), chemically reduced with ascorbic acid (▸), chemically reduced with ethylene glycol (▾), microwave-reduced under vacuum (•), thermally reduced between 0 and 2,000 °C (□) data interpolated with permission from^25^, chemically reduced with hydrazine (◻) data interpolated with permission from^26^, chemically reduced with hydrazine (⚫) data interpolated with permission from^27^, chemically reduced with hydrazine (▴) data interpolated with permission from^28^, (◀) graphene sample assuming <0.1% oxygen, (**c**) the variation in the I_D_/I_Gapp_ ratio as a function of the C/O ratio for the same samples.

The D’ peak is present in all defective graphenes and is therefore attractive as a measure of quality. However, due to the superposition of the G and D’ modes (giving rise to an apparent G peak, the G_app_), it is impractical to measure the position or intensity of the D’ mode. The second order transition (2D’, observed at ~3,200 cm^−1^) does not coincide with other modes and is an allowed mode even without defects, in the same way that the 2D is, and so can be observed even in pristine graphene. After GO is reduced the 2D’ peak occurs at higher energy, shifting by as much as 40 cm^−1^ from GO to graphene^19^, therefore, the difference between the 2D’ and the apparent G (G_app_) positions (2D’ − G_app_) increases. Also, by simply halving the energy of the 2D’ mode we can get the energy of the inferred D’ mode (D’_inf_) as it is not expected to vary as greatly as the 2D and D modes can^6^. The difference (D’_inf_  − G_app_) is more reliable than the absolute 2D’ or D’_inf_ position, which is subject to natural variation within samples, laser spot locations and measurement conditions. Figure 1(a,b) shows this clear relationship between the energy difference and the C/O atom ratio, as determined by X-ray photoelectron spectroscopy (XPS) or elemental analysis, for samples of rGO produced using various reduction methods and for pristine monolayer graphene (whose Raman spectrum displays no D mode). In contrast, the I_D_/I_Gapp_ ratio is not clearly correlated to the C/O atom ratio for the same samples (Fig. 1c), illustrating the difficulty in using the I_D_/I_G_ ratio across the full range of graphenes.

The correlation of the D’_inf_  − G_app_ with the C/O ratio also allows us to quantitatively describe the previously ill-defined boundaries between GO, rGO and graphene. We can define the three types of graphene derivatives by defining two points along the curve that mark the boundaries for three regions (see Fig. 1b):


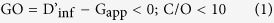



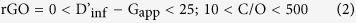



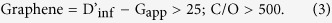


Considering the unreliability of the I_D_/I_G_ –structure relationship for GO and rGO, it is apparent that the contribution of the D’ mode to G_app_ had not been appropriately taken into account. In GO, the peak being measured as the G peak is actually the superposition of two peaks, the G and D’ peaks. The latter is a defect-derived peak that is observed in graphene at ~1,620 cm^–1 ^19,20. In graphite, the intensity of the D’ peak is proportional to the crystallite size, and hence the amount of defects^21^. For GO and rGO, which have comparatively high defect densities, the D’ peak is therefore expected to be much more intense, and thus contribute significantly to the G_app_ peak. In the Raman spectra of graphene samples, even those that have been subjected to ion bombardment to introduce lattice vacancies, the D’ peak remains distinct from the G peak^22^, but the properties and spectral signature of defective graphene do not closely approach those of even highly reduced GO. Ferrari and Basko predicted that the high defect density present in GO and rGO would produce D’ peaks whose energy was low enough to coincide with the G mode at ~1,600 cm^−1 ^6. The half width at half maximum above the G_app_ centroid position is larger than expected for many GO samples, implying that the G mode is accompanied by an extra peak at slightly higher energy^15^. Further increasing the density of defects is predicted to give D’ modes at energies as low 1,580 cm^−1^, which would theoretically make it appear at lower energy than the G peak. The origins of this large shift in the energy of the D’ mode remains unclear, but could depend on the second-nearest neighbour force constants^19^. The impact of such a large position shift on the position and intensity of G_app_ has not been explored.

**Figure 2:**
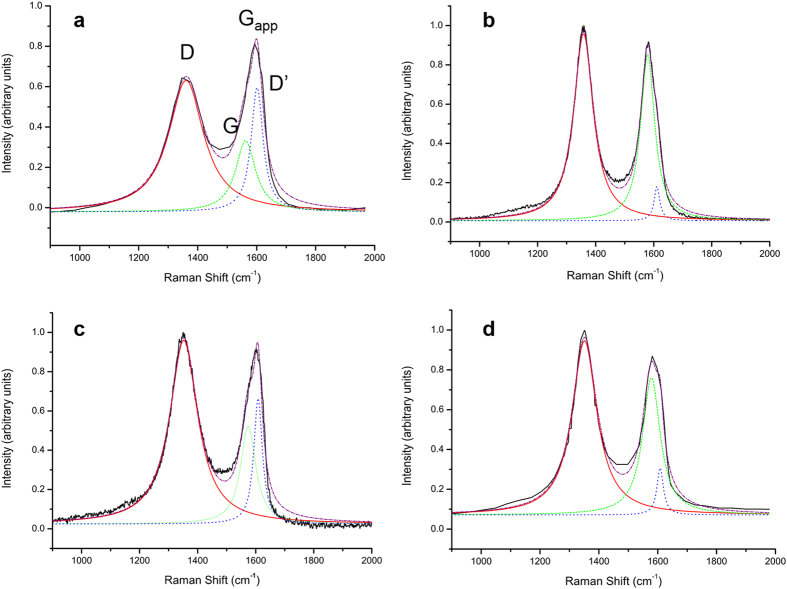
Example of two-peak fits applied to the G_app_ peak of GO and rGO. (**a**,**b**) GO before and after thermal reduction at 1,000 °C respectively and (**c**,**d**) GO before and after reduction with hydrazine, respectively (modified with permission from^16^).

When the peak at ~1,600 cm^−1^ is understood as arising from two modes, rather than interpreted as a single mode (Fig. 2), the observed peak positions and intensities are redefined, and the spectral differences between GO and rGO are clarified. Upon GO reduction, the position of the G peak increases and both the intensity of D’ and the I_D_/I_G_ ratio decrease, all of which are expected consequences of greater graphitisation following reduction (Table 1). The change in I_D_/I_G_ is particularly important, as interpreting the spectra in Fig. 1 based upon a single G_app_ peak would produce an increase in the ratio rather than a decrease, as was reported by Stankovich *et al*.^16^. This peak overlap greatly limits the utility of I_D_/I_G_ as a measure of defect density in GO and rGO.

**Figure 3:**
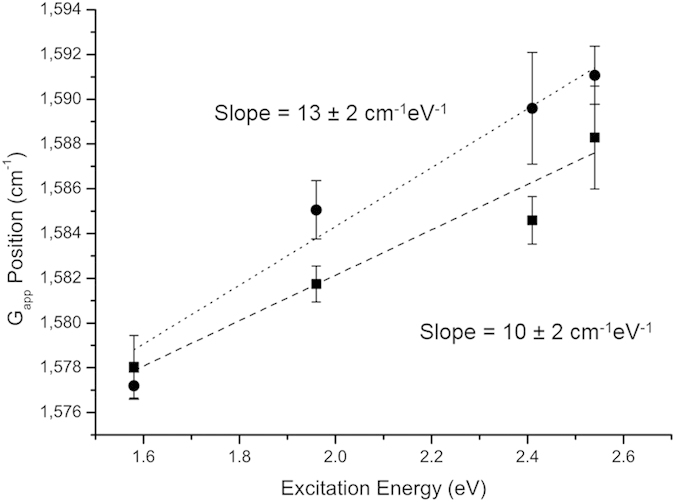
Dispersion of G_app_ position with changing excitation energy for GO (•) and an rGO reduced under nitrogen at 600 °C ( □), with the line of best fit and gradient for each, standard error in the mean used for error bars and least squares linear regression for the line of best fit.

Excitation-energy-dependent position shifts (dispersion) of the G_app_ peak have been measured in graphene samples with a high density of lattice vacancies to be ~6 cm^−1^eV^−1 ^22. This dispersion has been predicted, without explanation, to apply to all highly defective nanocrystalline graphites^6^. We contend that the dispersion of the G_app_ peak is another artefact of the superposition of a nondispersive G and an intense and dispersive D’ peak. The D’ peak has well-described dispersive behaviour; whereas the G peak has no known mechanism for dispersion. Pimenta *et al*. measured the dispersion of the D’ peak to be 10 cm^−1^eV^−1^ in graphene^23^, this is the same dispersion we measured for the G_app_ peak of rGO (Fig. 3). The dispersion was slightly higher (13 ± 2 cm^−1^eV^−1^) for the as-produced GO sample (i.e. the most defective material), allowing for some minor dispersion in the actual G peak. Thus, the net dispersion of G_app_ is most likely due to the dispersion of the D’ peak.

The peak commonly labelled ’G’ in the Raman spectra of GO and rGO samples is in fact a superposition of the G and D’ peaks (the G_app_ peak). This superposition renders the I_D_/I_Gapp_ ratio an unreliable measure for the reduction of GO, and has prevented it from accurately describing some of the recently reported GO and rGO samples. We propose that the best measure of GO reduction is the difference in the positions of the D’_inf_ and G_app_ peaks, i.e. D’_inf_  − G_app_ or the equivalent 2D’ − G_app_. The strong correlation that exists between the C/O atom ratio and D’_inf_ − G_app_, illustrated for a range of GO and rGO samples from different laboratories and using different reduction techniques (Fig. 1), demonstrates the value of this simple and robust tool for the analysis of the degree of reduction in GO. This correlation also provides for the quantified definition of GO, rGO and graphene based upon a simple and rapid Raman spectroscopic measure.

In addition to demonstrating a superior methodology for the characterisation of graphene materials, we have shown for practical purposes we can define three regions of reduction: A material with D’_inf_ − G_app_ < 0 can be defined as GO, one with 0 < D’_inf_ − G_app_ <25 is defined as rGO and samples with D’_inf_ − G_app_ > 25 can be defined as graphene. We have shown that the D’ and 2D’ modes, which have been overlooked in the Raman spectra of GO and rGO, provide valuable insight into all defective graphene materials. The amount of reduction can be predicted from the energy difference between the G_app_ and D’ or 2D’ peaks. We suggest that the consideration of the second-order Raman modes is vital to fully and accurately characterise graphene and its derivatives.

## Experimental

### Spectroscopy and analysis

All Raman spectroscopy was performed on an in Via Renishaw Raman spectroscope, using dry samples on a glass substrate. A 50x objective was used for all measurements and the system was used unpolarised with a 514 nm argon ion laser unless otherwise stated. The laser power was reduced to avoid sample damage; thus 1% power was used at 488 nm, 5% at 514 nm and 10% at 633 and 785 nm. All spectra were gathered over 10 s exposures and 1 (488 nm and 514 nm) or 2 accumulations (633 nm and 785 nm). For spectral analysis each peak was fitted to a single Lorentzian (except in Fig. 2 and Table 1 as explicitly described) using the standard fitting functions within Origin Pro 8 using the automatic parameter initialization, the fits all achieved an R^2^ greater than 0.98. The four spectra presented in Fig. 2 and Table 1 used a two-Lorentzian peak fit for the G_app_ peak, using the same software. The Raman spectra taken from the literature were interpolated using GraphClick software and then analysed as above. XPS analysis was carried out on an ESCALAB250Xi Thermoscientific spectroscope, using focused monochromated Al Kα radiation. The spectra were fitted using Avantage software.

### Reduction of GO

GO samples were synthesised using the modified Hummers method as described in^9^,^24^. The synthesised materials were washed in water with centrifugation, this process being repeated until the solution reached neutral pH. GO was reduced using two primary techniques: thermal and chemical. Two types of thermal reduction were used. The first was reduction in a furnace under nitrogen flow at 400, 600 and 800 °C for 4 h and 1,000 °C for 2 h, with a ramp rate of 4 °C min^−1^. The second was performed in a commercial microwave under vacuum (4 × 10^−4^ mbar) for 2 or 6 s. For the chemical reduction ascorbic acid was mixed with GO in a ratio of 10:1 by mass, and stirred at 85 °C for ~24 h until the reaction reached completion, then heated in the microwave at 150 °C for 30 min (ramp rate 1 °C min^−1^). The same process was used for reductions with ethylene glycol. Samples of 1 mgml^−1^ GO in ethylene glycol were prepared and diluted with twice as much deionised water by volume prior to reduction as above. Pristine single layer graphene was purchased and used as is from Graphenea.

## Additional Information

**How to cite this article**: King, A. A. K. *et al*. A New Raman Metric for the Characterisation of Graphene oxide and its Derivatives. *Sci. Rep*. **6**, 19491; doi: 10.1038/srep19491 (2016).

## Figures and Tables

**Table 1:** Spectral features from a two-peak fit of the G_app_ band from Fig. 2.

Figure 2 Sample	G position	I_D_/I_G_	D’ Intensity
a GO-1	1,574.2	1.88	0.82
b rGO-1	1,579.0	1.12	0.22
c GO-2	1,574.0	1.42	0.49
d rGO-2	1,579.0	1.24	0.27
